# Comparison of breast and bowel cancer screening uptake patterns in a common cohort of South Asian women in England

**DOI:** 10.1186/1472-6963-10-103

**Published:** 2010-04-27

**Authors:** Charlotte L Price, Ala K Szczepura, Anil K Gumber, Julietta Patnick

**Affiliations:** 1 Warwick Medical School, University of Warwick, Coventry, UK; 2 NHS Cancer Screening Programmes, Fulwood House, Old Fulwood Road Sheffield, UK

## Abstract

**Background:**

Inequalities in uptake of cancer screening by ethnic minority populations are well documented in a number of international studies. However, most studies to date have explored screening uptake for a single cancer only. This paper compares breast and bowel cancer screening uptake for a cohort of South Asian women invited to undertake both, and similarly investigates these women's breast cancer screening behaviour over a period of fifteen years.

**Methods:**

Screening data for rounds 1, 2 and 5 (1989-2004) of the NHS breast cancer screening programme and for round 1 of the NHS bowel screening pilot (2000-2002) were obtained for women aged 50-69 resident in the English bowel screening pilot site, Coventry and Warwickshire, who had been invited to undertake breast and bowel cancer screening in the period 2000-2002. Breast and bowel cancer screening uptake levels were calculated and compared using the chi-squared test.

**Results:**

72,566 women were invited to breast and bowel cancer screening after exclusions. Of these, 3,539 were South Asian and 69,027 non-Asian; 18,730 had been invited to mammography over the previous fifteen years (rounds 1 to 5). South Asian women were significantly less likely to undertake both breast and bowel cancer screening; 29.9% (n = 1,057) compared to 59.4% (n = 40,969) for non-Asians (p < 0.001). Women in both groups who consistently chose to undertake breast cancer screening in rounds 1, 2 and 5 were more likely to complete round 1 bowel cancer screening. However, the likelihood of completion of bowel cancer screening was still significantly lower for South Asians; 49.5% vs. 82.3% for non-Asians, p < 0.001. South Asian women who undertook breast cancer screening in only one round were no more likely to complete bowel cancer screening than those who decided against breast cancer screening in all three rounds. In contrast, similar women in the non-Asian population had an increased likelihood of completing the new bowel cancer screening test. The likelihood of continued uptake of mammography after undertaking screening in round 1 differed between South Asian religio-linguistic groups. Noticeably, women in the Muslim population were less likely to continue to participate in mammography than those in other South Asian groups.

**Conclusions:**

Culturally appropriate targeted interventions are required to reduce observed disparities in cancer screening uptakes.

## Background

Breast and bowel cancer are the two most common cancers in women in the United Kingdom (UK) [1]. In response to the recommendations of an expert working group [2], the National Health Service (NHS) established a population screening programme for breast cancer over 20 years ago in 1988. All women aged 50-70 years registered with a general practitioner (GP) are now routinely invited to undertake mammography every three years.

In 2000, the Department of Health set up a UK pilot to assess the feasibility of a national screening programme for bowel cancer [3]. Five year relative survival rates for patients diagnosed with colon or rectal (bowel) cancers in England and Wales were acknowledged to be poor; 53% and 52% respectively for females and 52% and 50% respectively for males in the period 2000 to 2001 [4]. Following the successful completion of the pilot, the NHS began rolling out a national screening programme based on a faecal occult blood test (FOBT) completed at home. All screening centres were open by the end of January 2010. During the pilot, men and women aged 50 to 69 were invited to undertake bowel cancer screening. However, the main programme is aimed at 60 to 69 year olds, which is the age group for whom the benefits of screening are expected to be largest. By the end of 2010, decisions will be taken about possible roll-out to people in their 50s [5]. All eligible men and women registered with a GP are now invited to undertake bowel cancer screening every two years.

Prior to roll-out of the national bowel cancer screening programme, a study was funded by the NHS Cancer Screening Programmes to investigate equity of bowel cancer screening uptake by ethnic minority populations in the English pilot site (Coventry and Warwickshire). This research revealed significantly lower uptake levels in the South Asian populations after correcting for socio-economic and other demographic differences [6]. Subsequent research has similarly identified continued lower mammography uptake among South Asian population groups fifteen years after the UK screening programme was introduced [7].

A number of international studies have reported inequalities in uptake of cancer screening services by minority ethnic populations [8-22]. Although lower uptakes have been reported for both breast and bowel cancer screening, research separating the influence of ethnicity from that of deprivation is rare, with such analyses almost exclusively based on research in the United States [15,23]. A further shortcoming of most studies to date is that they explore ethnic differences in screening uptake for a single cancer only. Research which compares the response of individuals to different cancer screening programmes is lacking.

This paper reports on an analysis of breast and bowel cancer screening uptake in a large cohort of women in England, with a focus on equity in uptake and access to the two existing national cancer screening programmes. Uptake patterns for breast cancer screening over a fifteen year period, including regularity of screening and the relationship between breast cancer screening behaviour and initial response to the new bowel screening programme, are also examined. This research is part of a broader programme commissioned by the NHS Cancer Screening Programmes which aims to improve the accessing of cancer screening by ethnic minority communities in the UK.

## Methods

### Setting

The study was undertaken in the Coventry and Warwickshire area (NHS bowel cancer screening pilot site for England). This covers a population of over 800,000, including 8.7% ethnic minority residents, mainly of South Asian origin [24]. This percentage is slightly higher than the 7.9% reported in 2001 for the UK as a whole.

### Data Sources

Breast cancer screening data were provided by the Warwickshire, Solihull and Coventry Breast Screening Service. Data on bowel cancer screening were obtained from the Screening Unit covering the area, based in Rugby. Data downloads for both programmes were limited to women resident in the area. Data covered rounds 1, 2 and 5 of the breast cancer screening programme (1989-1992, 1992-1995 and 2001-2004 respectively) and round 1 of the bowel cancer screening pilot (2000-2002).

### Data Preparation

All women were identified who had been invited to undertake both breast and bowel cancer screening during the period 2000-2002 (a common cohort). A subset of women in this cohort invited to undertake breast cancer screening over a period of fifteen years (rounds 1, 2 and 5) was also identified. Data for both programmes were collated at individual invitee level to include NHS number (identifier), demographic descriptors, invitation date, screening uptake, subsequent assessments and diagnostic outcome.

Women were removed from the data if they: (i) had no NHS number recorded, (ii) had been excluded from either type of screening by the Health Authority (e.g. undergoing treatment or recently deceased), or (iii) were outside of the specified age range (50-69 years). For women who received two invitations to breast cancer screening during the period corresponding to round 1 of the bowel cancer screening pilot, only the earlier record was retained. Individuals were matched across breast and bowel cancer screening data sets using their NHS number.

Routine ethnic monitoring data is not available in England for those registered in the NHS. Detailed information is collected in the Census (see Table 1), but this cannot be linked to individuals. Although there has been a steady growth in the recording of patient ethnicity in hospital trusts since 1996, a recent study has identified poor recording in relation to cancer [25]. In primary care, financial incentives have been introduced to encourage GPs to record the ethnic group of all new patients [26]. For these reasons, name recognition software, *Nam Pehchan*, for which sensitivity and specificity values of 95% and 97% respectively have been reported [27,28], was used to assign an ethnicity label to each woman. The software matches against the complete name (or the name stem) to determine whether a person is of South Asian origin and attaches appropriate language and religion markers. Using this information, women were placed into one of six religio-linguistic groups: non-Asian; Hindu-Gujarati; Hindu-Other; Muslim; Sikh; South Asian-Other. Characteristics of the South Asian groups are shown in Table 2. The software dictionary was refined further through manual checking of all names for residents in the area, producing an estimated final sensitivity of 97%. Throughout this paper, the five South Asian religio-linguistic groups are referred to collectively as 'South Asian'.

**Table 1:** Categories of ethnic group recorded in the UK Censuses of 1991 and 2001

1991	2001
White	White - British

	White - Irish

	White - Any other White background (please write in)

(Other...)	Mixed - White/Black Caribbean

	Mixed - White/Black African

	Mixed - White/Asian

	Any other mixed background (please write in)

Black- Caribbean	Black or Black British:
	Caribbean

Black- African	Black or Black British:
	African

Black- Other (Please describe)	Black or Black British:
	Any other background (please write in)

Indian	Asian or Asian British
	Indian

Pakistani	Asian or Asian British
	Pakistani

Bangladeshi	Asian or Asian British
	Bangladeshi

Asian- Other (Please describe)	Asian or Asian British
	Any other background: (please write in)

Chinese	Chinese or Other Ethnic group
	Chinese

Any Other Ethnic Group (Please describe).	Chinese or Other Ethnic group
	Any other: (please write in)

**Table 2:** Characteristics of ethnic groups identified by Nam Pehchan

	Characteristics				
	
Ethnic Group	% Population 50-69 yrs Born Abroad	Language	Religion	Diet	Literacy
				
				Male Female	Male Female
1. Hindu-Gujerati				M: Vegetarian	M: Good
	>95%	Gujerati	Hinduism	F: Vegetarian	F: Fair

2. Hindu-Other		Hindi/		M: Mostly veg	M: Fair
	>95%	Bengali	Hinduism	F: Vegetarian	F: Poor

3. Muslim				M: Non-veg*	M: Fair-poor
	>90%	Urdu	Islam	F: Non-veg*	F: Very low

4. Sikh				M: Non-veg*	M: Fair
	>90%	Punjabi	Sikhism	F: Vegetarian	F: Poor

5. Other Asian				M: Mostly veg	M: n/a
	>95%	Mixed	Mixed	F: Mostly veg	F: n/a

* Diet includes red meat

### Analysis

Breast and bowel cancer screening uptake levels for each ethnic group were calculated and compared using the chi-squared test. For breast cancer screening, uptake was defined as completion of a mammogram in response to a routine invitation. For bowel cancer screening, uptake was defined as the satisfactory completion of an FOBT home kit resulting in a laboratory result. Patterns of uptake behaviour in the common cohort were compared. A more detailed description of the study methods is reported elsewhere [7].

## Results

**Figure 1:**
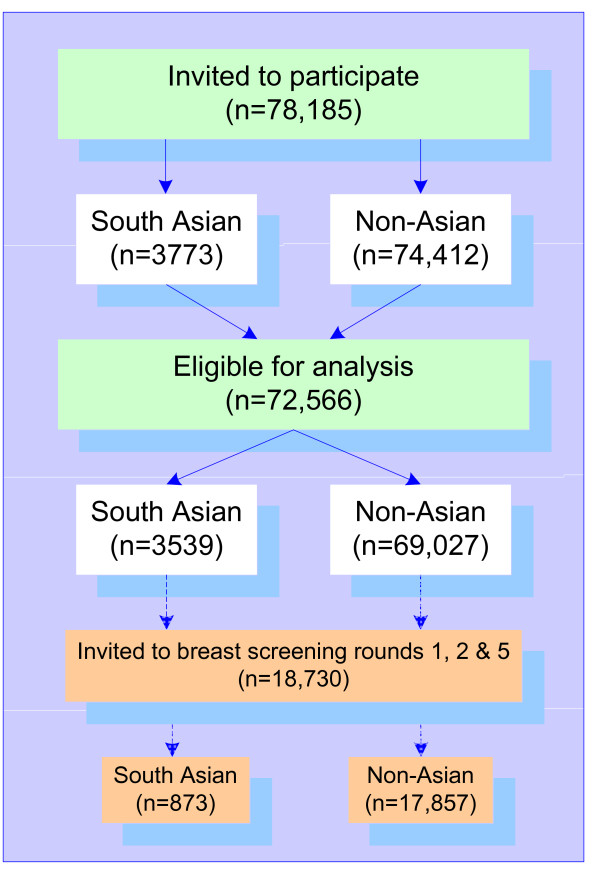
**Flow diagram of common cohort**.

A total of 78,185 women were identified who had been invited to undertake both breast and bowel cancer screening during the period 2000-02 (Figure 1). After exclusions, this common cohort consisted of 72,566 women; 3,539 South Asian and 69,027 non-Asian. A subset of 18,730 women who had been invited to undertake breast cancer screening in rounds 1, 2 and 5 (1989-2004) was extracted; 873 South Asian and 17,857 non-Asian.

### Response to breast screening versus bowel screening in same period

Table 3 shows that although 86% (*n *= 62,425) of women completed at least one form of cancer screening during 2000-02, this figure was significantly lower for South-Asians than non-Asians; 74.3% (*n *= 2,630) vs. 86.6% (*n *= 59,795), *p *< 0.001. The likelihood that an individual would undertake both forms of screening was also far lower among South Asians; 29.9% (*n *= 1,057) vs. 59.4% (*n *= 40,969), *p *< 0.001, and a significantly higher proportion of South Asian women completed only one type of screening during this period; 44%, *n *= 1,573 vs. 27.3%, n = 18,826, *p *< 0.001. A decision to participate in the new bowel cancer screening programme was less likely overall, particularly among South Asians.

**Table 3:** Uptake of breast and bowel screening by ethnicity (2000-02); numbers (row percentages)

Ethnicity	Breast and bowel screening uptake								
	
	Breast & bowel		Breast only		Bowel only		Neither		Total
	**Count**	**(%)**	**Count**	**(%)**	**Count**	**(%)**	**Count**	**(%)**	**Count**

South Asian	1057	(29.9)	1430	(40.4)	143	(4.0)	909	(25.7)	3539

Non-Asian	40969	(59.4)	14091	(20.4)	4735	(6.9)	9232	(13.4)	69027

Hindu-Gujarati	230	(35.8)	267	(41.6)	22	(3.4)	123	(19.2)	642

Hindu-Other	113	(32.3)	144	(41.1)	12	(3.4)	81	(23.1)	350

Muslim	164	(22.3)	238	(32.3)	37	(5.0)	297	(40.4)	736

Sikh	534	(30.4)	762	(43.4)	67	(3.8)	391	(22.3)	1754

South Asian Other	16	(28.1)	19	(33.3)	5	(8.8)	17	(29.8)	57

Total	42026	(57.9)	15521	(21.4)	4878	(6.7)	10141	(14.0)	72566

Examination of religio-linguistic groups in the South Asian cohort revealed differences in behaviour patterns. For all except one group (Muslim women), the most common behaviour was to accept breast cancer screening only. In the Muslim group, refusal of both types of screening was the most likely response. In contrast, the most common response for non-Asian women was to accept both types of cancer screening.

### Response to breast cancer screening over time versus new bowel cancer screening programme

**Figure 2:**
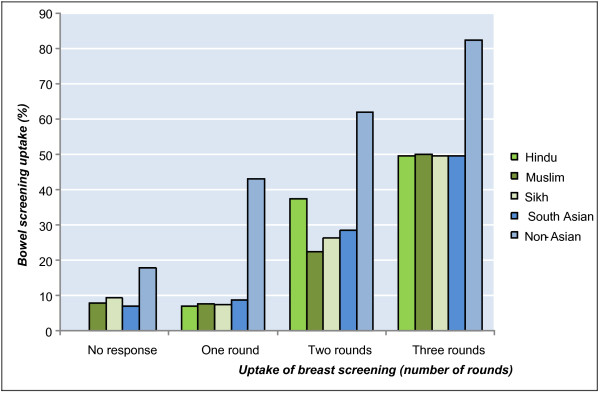
**Completion of bowel cancer screening by breast cancer screening uptake (rounds 1, 2 and 5)**.

In the subset of women whose breast cancer screening behaviour was recorded over fifteen years, successful completion of the FOBT kit was more likely among those who had consistently chosen to participate in breast cancer screening (Figure 2). For the non-Asian group, over four out of five women who underwent mammography in all three rounds also successfully completed an FOBT home kit (82.3%). The comparable figure was far lower in the South Asian group (49.5%, p < 0.001).

Figure 2 shows that, in the non-Asian group, even if a woman only undertook breast cancer screening in one round, the likelihood of successfully returning an FOBT kit was higher than for a woman who opted out of all three breast cancer screening rounds (43.1% vs. 17.9%, p < 0.001). In contrast, a South Asian woman who undertook breast cancer screening in only one round was no more likely to complete bowel cancer screening than one who opted out altogether (8.7% vs. 7%, p = 0.228), with women in both groups much less likely to complete bowel cancer screening than women in the non-Asian group.

### Maintaining breast cancer screening behaviour over time

Table 4 presents uptake results for the subset of women whose breast cancer screening behaviour was recorded over a fifteen year period. In both rounds 2 and 5, mammography uptake was highest for women (South Asian and non-Asian) who had chosen to undertake breast cancer screening at the outset of the programme (i.e. round 1); 82% and 74.1% for South Asians, and 91.8% and 85.3% for non-Asians respectively. Uptake in round 5 was much lower, and virtually identical, for women in both groups who had refused screening in round 1; 38.8% for South Asians and 39.1% for non-Asians.

**Table 4:** Breast screening uptake over time by ethnicity; numbers (row percentages)

		Subsequent screening uptake (number screened: percentage)								
		
Ethnicity	Screened: round 1	Round 2		Round 5		Round 2 or 5		Rounds 2 & 5		Total
			
		Count	(%)	Count	(%)	Count	(%)	Count	(%)	
South Asian	Yes	532	(82.0)	481	(74.1)	591	(91.1)	422	(65.0)	649
	
	No	93	(41.5)	87	(38.8)	124	(55.4)	56	(25.0)	224

Non-Asian	Yes	13418	(91.8)	12471	(85.3)	14069	(96.2)	11820	(80.9)	14618
	
	No	1187	(36.6)	1266	(39.1)	1571	(48.5)	882	(27.2)	3239

Hindu- Gujarati	Yes	109	(83.2)	103	(78.6)	121	(92.4)	91	(69.5)	131
	
	No	14	(41.2)	15	(44.1)	20	(58.8)	9	(26.5)	34

Muslim	Yes	99	(79.2)	76	(60.8)	107	(85.6)	68	(54.4)	125
	
	No	24	(34.3)	18	(25.7)	32	(45.7)	10	(14.3)	70

Sikh	Yes	275	(82.1)	257	(76.7)	308	(91.9)	224	(66.9)	335
	
	No	45	(44.6)	43	(42.6)	58	(57.4)	30	(29.7)	101

Total	Yes	13950	(91.4)	12952	(84.8)	14660	(96.0)	12242	(80.2)	15267
	
	No	1280	(37.0)	1353	(39.1)	1695	(48.9)	938	(27.1)	3463

However, an initial reluctance to undertake breast cancer screening did not necessarily remain fixed over time, with 55.4% of South Asians and 48.5% of non-Asians who refused screening in round 1 subsequently accepting at least one invitation to mammography. At the same time, consistency in the decision to undertake screening was far less apparent in the South Asian population, with only 65% of South Asians vs. 80.9% of non-Asians undertaking mammography in rounds 2 and 5 after accepting screening in round 1 (p < 0.001). This effect was particularly noticeable in Muslim women, where only half (54.4%) of those who underwent mammography in round 1 continued to accept breast cancer screening in rounds 2 and 5.

## Discussion

A key challenge for any public health programme is engagement with its target populations. Since the UK now advocates population screening for breast and bowel cancer, and one in twelve UK residents are from a black or minority ethnic (BME) background, the response of these populations to both cancer screening programmes will have an important impact on their effectiveness and cost-effectiveness [29,30]. The NHS Cancer Reform Strategy therefore encourages primary care trusts (PCTs) to use targeted strategies to increase participation in screening by BME communities. However, to do this, PCTs need improved evidence on local uptake levels and, in particular, on how a person's ethnicity affects response.

The present study confirms evidence from earlier research reporting low cancer screening uptake in South Asian populations [11,13,18,31,32]. However, UK studies have mostly estimated screening patterns using uptake figures for a geographical area or general practice and by comparing these to Census data for the same population, rather than examining individual patient behaviour. This is primarily due to the poor state of ethnicity recording in NHS primary care [33] and, in particular, poor recording in relation to cancer [25]. The present study has been able to examine the behaviour of individuals by using name recognition software.

Overall, our findings show that for both types of screening programme, South Asian women were more likely to decide not to participate, with the largest disparity recorded for bowel cancer screening. The most common behaviour pattern observed in the majority non-Asian population was for a woman to undertake both breast and bowel cancer screening (59.4%), while South Asian women were most likely to undertake breast cancer screening only (40.4%). Our findings show that uptake of cancer screening is particularly low in the Muslim population, with four out of ten Muslim women deciding to opt out of both breast and bowel cancer screening. There also appears to be a difficulty in ensuring continuity of uptake in the South Asian population, especially among Muslim women. Global figures which show a decreasing gap in breast cancer screening uptake over time between South Asian women and the majority population [7] might therefore mask differences in the continuity of uptake [34]. Just under half (45.6%) of Muslim women who undertook mammography in round 1 decided not to participate in both of rounds 2 and 5, mainly choosing instead to participate in just one of the two subsequent rounds.

In terms of evidence to support cancer screening, it is estimated that reduction in mortality from breast cancer over a 10-year period after first invitation for screening would be 0.9 per 1000 for women aged 50-59 [35]. Similarly, a large-scale UK based study reported a 13% reduction in mortality from colorectal cancer associated with faecal occult blood screening (95% CI: 3-22%) [36]. Furthermore, the 2007 Cochrane Review on screening for colorectal cancer has estimated the relative reduction in mortality from repeated bowel cancer screening as 16% and concluded that the benefits of colorectal screening include a modest reduction in mortality [37]. However, similar figures are not available for the South Asian population, although the incidence of many cancers is thought to be lower in these groups [38].

Health promotion materials must therefore communicate the importance and benefits of regular screening, whilst also outlining the potential harms in a culturally sensitive manner to allow South Asian women to make an informed decision about participation [35]. The most common causes of harm from screening are through false positive results which lead to unnecessary investigations and anxiety, and through over-diagnosis (i.e. the detection of cancers that were not destined to cause death or symptoms) [39]. According to Morrison [40], both harms are inevitable if a screening programme is to be effective although, once again, levels are not known for UK South Asian populations.

Longer term screening behaviour of South Asian women who participated in the first round of the new NHS bowel cancer screening programme should ideally be monitored. The present study provides evidence from breast cancer screening that although an initial decision to undertake screening increases the likelihood of accepting a second (bowel) screening programme, the effect is less pronounced for South Asian women. This suggests that South Asian women's needs might best be addressed through more targeted approaches for bowel cancer screening. Strategies may include targeting women who have opted out of screening (i.e. non-responders) in order to raise awareness of the potential health benefits, and targeting generic information at first time invitees [41]. Our findings suggest that women who participated in breast cancer screening at initial invitation in the first round were more likely attend for screening in subsequent rounds (see Table 4), thus suggesting the importance of maximising uptake at the earliest stage. Furthermore, name recognition software such as *Nam Pehchan *could be used to prospectively identify the ethnicity of specific groups in order to provide tailored information.

South Asian women who undertook breast cancer screening once were no more likely to complete the FOBT home kit than those who decided against it altogether in rounds 1, 2 and 5. As the first home-based UK cancer screening test, it is perhaps not unexpected that FOBT kits might present greater barriers for ethnic minority women. Home testing requires that an individual understand the benefits of screening and can follow written instructions in order to collect and preserve samples. The evaluation of the UK Colorectal Screening Pilot Programme by ethnicity showed that South Asians were more likely to require more than one FOBT home kit in order to obtain a valid result, with a large proportion requiring four or more kits [6]. South Asian women generally have poorer literacy, with Muslim women showing particularly low levels [42,43], which may explain the poor uptake in this population. Non-written information may be more appropriate for this group. Breast and bowel cancer screening CDs [44,45] and DVDs [46,47] have been produced in various languages for the South Asian community; these still need to be evaluated in a routine NHS service setting.

A review of the literature on interventions aimed at improving breast cancer screening uptake by ethnic minority populations concluded that a combination of strategies to reduce barriers and enhance access, focused on individuals and system-wide, is most effective [48]. At present, there is insufficient research evidence to identify which types of intervention are best for increasing bowel cancer screening uptake by BME populations [49-52]. However, US studies show that active physician encouragement is an important influence for improving bowel cancer screening uptake by minorities [53,54]. Our findings also suggest that a potential strategy could involve targeting South Asian women at first invitation and monitoring their compliance over time, with own language materials emphasising the importance of continuity in cancer screening programmes. Specific sub-groups (e.g. Muslim women) should be high priority for such targeted interventions in order to achieve equity in uptake.

This study was unable to draw conclusions about the African Caribbean population due to incomplete ethnic monitoring data and the fact that the software used is name-based and cannot identify these individuals. However, the African-Caribbean population is much smaller and geographically dispersed, the latter making socio-economic status difficult to assess based on Census data. Furthermore, evidence from other UK cancer screening programmes indicates that African-Caribbean uptake rates are close to the population mean [55]. The study was also unable to examine the influence of additional ethnic factors, such as language spoken at home, cultural background and country of origin, due to the lack of ethnic monitoring data in the UK. However, the value of at least some of this information is of limited use. It has been shown that country of origin provides a poor indicator of ethnicity due to the increasing number of people in this group who are born in the UK [56].

## Conclusions

There is a need to identify and assess culturally appropriate interventions to reduce observed differences in cancer screening uptake. This should include provision of tailored evidence-based health promotion materials for South Asian subgroups which will allow invitees to make an informed decision about cancer screening. More detailed examination of behaviour across screening programmes could help to identify women who, having made the decision to undertake breast cancer screening, might be encouraged to also undertake bowel cancer screening.

## Competing interests

The authors declare that they have no competing interests.

## Authors' contributions

AKS and JP conceived the idea for the study. AKG obtained the data. CLP cleaned the data and completed the statistical analysis. CLP and AKS wrote the manuscript which was reviewed by all authors. All authors had full access to all data in the study and can take responsibility for the integrity of the data and the accuracy of the data analysis. All authors read and approved the final manuscript.

## Pre-publication history

The pre-publication history for this paper can be accessed here:

http://www.biomedcentral.com/1472-6963/10/103/prepub
